# Supplementary figures and images for: Children and young people’s beliefs about mental health and illness in Indonesia: A qualitative study informed by the Common Sense Model of Self-Regulation
Source: PLoS One. 2022 Feb 4;17(2):e0263232. doi: 10.1371/journal.pone.0263232 (PMC8815881; doi:10.1371/journal.pone.0263232)

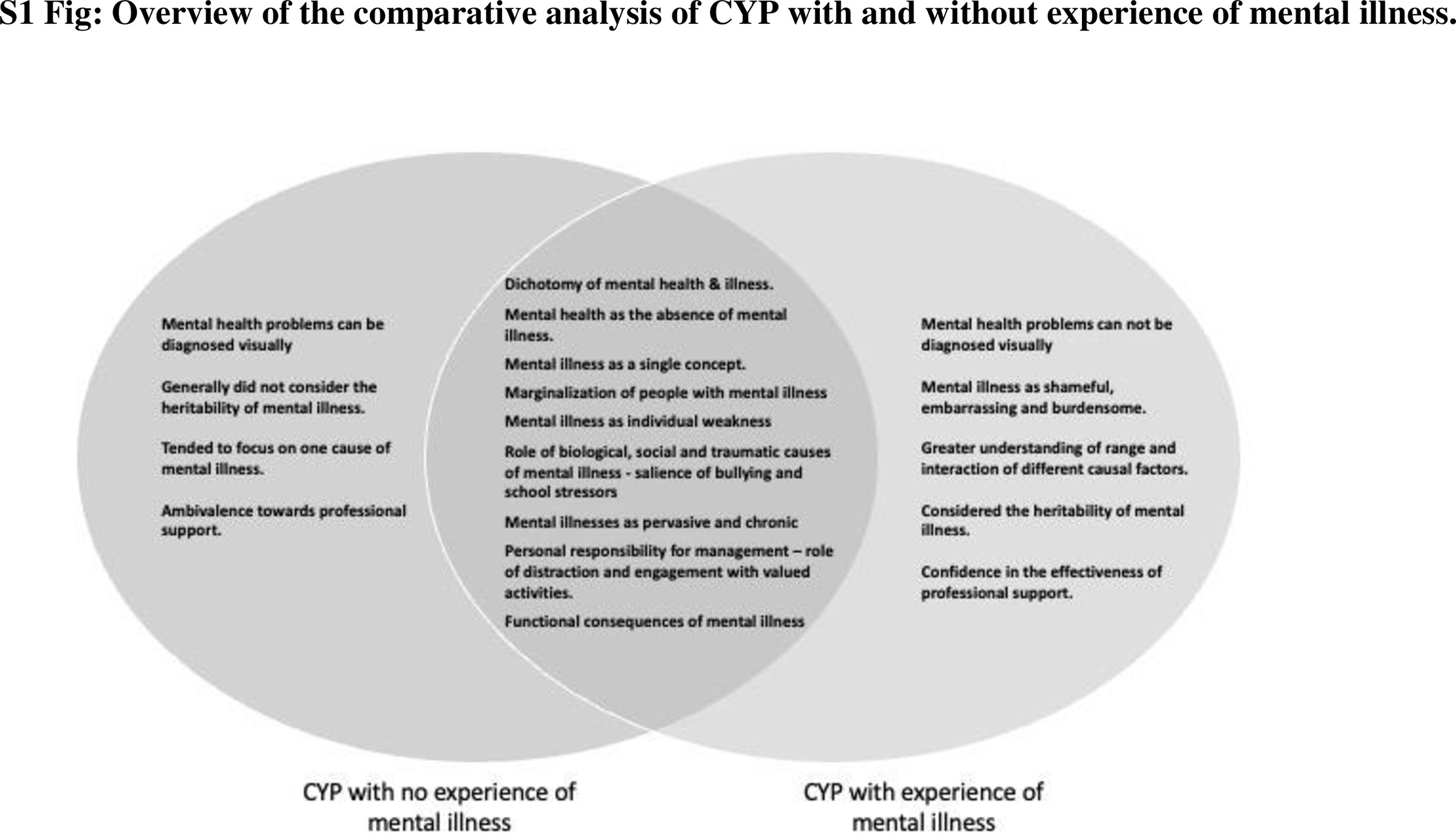

Supplement: S1 Fig — (JPG) [file pone.0263232.s001.jpg]
