# Supplementary material for: Children and young people’s beliefs about mental health and illness in Indonesia: A qualitative study informed by the Common Sense Model of Self-Regulation
Source: PLoS One. 2022 Feb 4;17(2):e0263232. doi: 10.1371/journal.pone.0263232 (PMC8815881; doi:10.1371/journal.pone.0263232)
Supplement: S1 File — (PDF) [file pone.0263232.s002.pdf]

Supporting File 1:

Photo crib sheet

An invitation….

We would like you to invite you to take some photographs to bring along to your next meeting with the researcher. We want the photographs to show **what mental health means to you.**

You don't have to take photographs if you don't want to – it is up to you.

How we will use the photographs

The researcher will talk to you about the photographs when you next meet. If you are taking part as part of a group, this might mean that other people in the group may see them. We know that using images and photographs can sometimes help young people to communicate.

We would like to display the photographs at an exhibition at the end of this study. This means that other people will see your photographs. Please only show something in the photograph that you are comfortable with others seeing. If you show anything in the photograph that we think identifies you (shows who you are) or another person (shows who they are), we will alter the photograph so no one can link the photograph to anyone.

A few tips to help:

- You can take just one photograph or a few, it is your choice
- When you meet with the researcher, they will ask you to tell them about what the photograph means
- The photograph can show anything that says something about mental health in your opinion – it can show an object, a place, an activity or a person.
- If your photograph shows a person, please remember to ask the person if they mind being in the photograph, as you normally would. It is important to tell the person that you are taking the photograph as part of a research study and that other people (researchers) might see it. Tell the person that the photographs might be shown at an exhibition, but only after the researchers have changed the photograph to hide anything that shows who they are.
- Please only submit photographs that you have taken yourself.

You can use your own camera or smartphone to take photographs or borrow one from us!

Using your own smartphone/camera

If you choose to use your own device, you must be happy to email the photographs to our team before you next meet the researcher. You might need to ask your parent/guardian to help you with this. You can send them to:
[insert researcher email address]

Using one of our smartphones

If you borrow one of our smartphone – just hand it back to the researcher when you meet. If you are taking part in the interview as part of a group, the researcher might arrange to collect the smartphone back from you before the group meeting. This is to allow the researcher to collect the photographs before the meeting.
