# Supplementary material for: Children and young people’s beliefs about mental health and illness in Indonesia: A qualitative study informed by the Common Sense Model of Self-Regulation
Source: PLoS One. 2022 Feb 4;17(2):e0263232. doi: 10.1371/journal.pone.0263232 (PMC8815881; doi:10.1371/journal.pone.0263232)
Supplement: Attachment — Submitted filename: PLOS One Impetus resonse to reviewers.docx [file pone.0263232.s003.docx]

Dr Bradford Dubik

Academic Editor
PLOS ONE

Wednesday, 3 November 2021

Dear Dr Dubik

**RE: PONE-D-21-04834. Children and young people's beliefs about mental health and illness in Indonesia: A qualitative study informed by the Common Sense Model of Self-Regulation**

Thank you for your email dated 2^nd^ October, for the helpful set of editorial and reviewer comments and the requested revisions. We have now the revised the manuscript in line with the comments from the reviewers which we feel has strengthened the manuscript. Please find below a point-by-point response to each comment detailing how we have addressed each point raised.

***Editorial comments***

**1. Please ensure that your manuscript meets PLOS ONE's style requirements, including those for file naming. The PLOS ONE style templates can be found at** 
[**https://journals.plos.org/plosone/s/file?id=wjVg/PLOSOne_formatting_sample_main_body.pdf**](https://journals.plos.org/plosone/s/file?id=wjVg/PLOSOne_formatting_sample_main_body.pdf)**and 
<https://journals.plos.org/plosone/s/file?id=ba62/PLOSOne_formatting_sample_title_authors_affiliations.pdf>**

We have now revisited the manuscript with the guidelines provided to ensure it meets PLOS ONE’s style requirements.

**2. Please note that in order to use the direct billing option the corresponding author must be affiliated with the chosen institute. Please either amend your manuscript to change the affiliation or corresponding author, or email us at** [**plosone@plos.org**](mailto:plosone@plos.org) **with a request to remove this option.**

The affiliation of the corresponding author is the University of Manchester which is detailed on the manuscript and aligns with the information on the editorial system. I have emailed the address provided for clarification.

**3. Please provide additional details regarding participant consent. In the Methods section, please ensure that you have specified (1) whether consent was informed and (2) what type you obtained (for instance, written or verbal). If your study included minors, state whether you obtained consent from parents or guardians. If the need for consent was waived by the ethics committee, please include this information.**

This information is provided in the data collection section on page 11. We have now specified this was informed consent.

*Interviews only proceeded if CYP provided informed verbal assent to take part and their parent/carer provided informed written consent for their child’s participation.*

**4. We note that Supporting information file 1 in your submission contain copyrighted images. All PLOS content is published under the Creative Commons Attribution License (CC BY 4.0), which means that the manuscript, images, and Supporting Information files will be freely available online, and any third party is permitted to access, download, copy, distribute, and use these materials in any way, even commercially, with proper attribution. For more information, see our copyright guidelines:** [**http://journals.plos.org/plosone/s/licenses-and-copyright**](http://journals.plos.org/plosone/s/licenses-and-copyright)**.**

**We require you to either (1) present written permission from the copyright holder to publish these figures specifically under the CC BY 4.0 license, or (2) remove the figures from your submission:**

**a. You may seek permission from the original copyright holder of the Figures in Supporting information file 1 to publish the content specifically under the CC BY 4.0 license.**

**We recommend that you contact the original copyright holder with the Content Permission Form (**[**http://journals.plos.org/plosone/s/file?id=7c09/content-permission-form.pdf**](http://journals.plos.org/plosone/s/file?id=7c09/content-permission-form.pdf)**) and the following text:
“I request permission for the open-access journal PLOSONE to publish XXX under the Creative Commons Attribution License (CCAL) CC BY 4.0 (**[**http://creativecommons.org/licenses/by/4.0/**](http://creativecommons.org/licenses/by/4.0/)**). Please be aware that this license allows unrestricted use and distribution, even commercially, by third parties. Please reply and provide explicit written permission to publish XXX under a CC BY license and complete the attached form.”**

**Please upload the completed Content Permission Form or other proof of granted permissions as an "Other" file with your submission.**

**In the figure caption of the copyrighted figure, please include the following text: “Reprinted from [ref] under a CC BY license, with permission from [name of publisher], original copyright [original copyright year].”**

**b. If you are unable to obtain permission from the original copyright holder to publish these figures under the CC BY 4.0 license or if the copyright holder’s requirements are incompatible with the CC BY 4.0 license, please either i) remove the figure or ii) supply a replacement figure that complies with the CC BY 4.0 license. Please check copyright information on all replacement figures and update the figure caption with source information. If applicable, please specify in the figure caption text when a figure is similar but not identical to the original image and is therefore for illustrative purposes only.**

We have removed the photographs from supporting document one.

**5. Please include captions for your Supporting Information files at the end of your manuscript, and update any in-text citations to match accordingly. Please see our Supporting Information guidelines for more information:** [**http://journals.plos.org/plosone/s/supporting-information**](http://journals.plos.org/plosone/s/supporting-information)**.**

Captions included for supporting information files at the end of the manuscript and in-text citations updated accordingly.

**Reviewer #1: - The introduction is somewhat lengthy. It is better to be a little brief.**

We have edited this down where possible but not extensively given that reviewers 2 and 3 felt that the introduction was a useful background to the study and requested additional material be included.

**- The methods section is well written and detailed.**

Noted.

**- Although the presented results are correct based on the model, but in general, it has limited the expression of the results. The tendency to provide comparisons between the two groups in expressing the results has reduced the general opinions of individuals and has limited the comparison of the two groups. Thematic analysis could, in addition to this analysis, show the general aspects of the participants' opinions.**

Thank you for this comment. We did not use the CSM in a purely deductive way rather in combination with inductive coding to ensure we did not lose the voice of the young people who participated in the study. Transcripts were coded inductively initially and then were mapped to the components of the CSM. No codes fell outwith the framework so we were reassured this accurately captured the experience of participants who took part in the study. We also presented our analysis to a patient and public involvement group who reviewed our framework and felt is adequately represents the experiences of mental health in Indonesia. We hope this reassures reviewer 1.

**- In expressing the results, there is no reference to the results of analysis by photo elicitation method.**

We have included detail in the methods section on page 13 that photographs provided by young people were not treated as a unit of data rather they were incorporated in the methodological approach in response to suggestions from our Indonesian partners that such activities would be important to ensure optimal engagement with participants given the sensitive nature of mental illness in Indonesia. References to provide support for the use of photo elicitation methods in this way are provided within the manuscript.

*Photographs provided by CYP were not treated as units of data but as tools to support CYP participation in interviews [34] and promote rapport between the interviewer and interviewee [35].*

**- The discussion section is well written and detailed.**

Noted.

**Reviewer #2: Introduction

The introduction does a good job laying the groundwork for the study, as far as the extent of the problem and the need to compliment the focus on structural barriers with individual-level considerations, such as illness perception.**

Noted. **This section, however, is missing the research by Munson and colleagues, who over a decade ago were the first team to apply the CSM to adolescents/young adults, and those with mood disorders. Authors need to acknowledge this work; please see Munson, Narendorf, Ben-David & Cole, 2019; Munson, Floersch, & Townsend, 2009. These studies provide context for this study as they are also exploring illness and treatment understanding/attitudes among adolescents.**

Thank you for this helpful suggestion. We have added this into the introduction and we agree they suggested papers help to provide further context for the current study. We have also included these papers in the discussion to further contextualise the findings of the current study. **Method

How did you code “experience of mental illness”? Was this based on child self-report? Or parent report? Or a screening measure? Or a medical record?**

Participants with experience of mental illness were recruited through child and adolescent mental health services in line with diagnoses of anxiety and depression. Only those with requisite levels of experience were approached to take part in the study. We have revised the manuscript to ensure this is now clear:

*CYP with a diagnosis of anxiety or depression were invited directly by child and adolescent mental health services. CYP with no experience of mental health problems were recruited through direct invitation by schools. Posters were also exhibited in relevant organisations to supplement the recruitment process and participants could contact the study team directly if they were interested in taking part in the study. However, all CYP were recruited through direct invitation.*

**Were the interview questions based on dimensions of the CSM, meaning was the study deductive, or was it inductive? Can the authors provide a sample question from the interview protocols to the reviewers/readers?**

We are sorry that this is not clear within the manuscript. We have now added detail to ensure this is prominent within the methods section and included examples of questions within the interview schedule.

*Interviews started with questions about the photographs they had taken and participants were asked to explain what they had meant to convey with their pictures and how this related to their understanding of mental health and illness. The remainder of the interview followed an interview schedule designed to elicit beliefs about positive mental health and wellbeing and mental illness. Questions explored CYP’s perceptions of mental health in general, with particular focus on anxiety and depression. Examplar questions included:*

- *What do you think ‘mental health’ means? (Prompt good mental health vs mental health problems).*
- *What do you think it means when people have a ‘mental health problem’ or ‘mental illness’?*
- *What do you think the signs of mental health problems are?*
- *What do you think the causes of mental health problems are?*
- *Is there anything that young people can do for themselves to look after their mental health?*
- *What sort of things can help people with mental health problems?*

**Sound, careful rigorous methods. No issues with the methods other than those indicated above.**

Noted. **Results

Emotional representations of illness, i.e., shame, is a dimension of the CSM. Why didn’t the authors consider some of the emotion data in relation to this construct?**

Thank you for this suggestion. We have separated the first construct to provide separate findings on identity beliefs and emotional representations of illness. **In the section on “cause of illness” you suggest that those with a mental illness were more likely to consider the “interaction of multiple factors”. This is an important finding, especially in contrast to the idea that individuals are inherently weak, which you suggest participants reported. Authors need to support this result of multi-factorial causality with an empirical data element. Generally, this paper needs more data included in the manuscript. This is an area where the authors should also compare these results to other studies, i.e., See Munson et al., 2019 for comparison “cause” data among older adolescents/young adults.**

We welcome the opportunity to include more quotes within our manuscript. We have included an additional quote to support the interaction of multiple factors interpretation and added additional quotes throughout the manuscript whilst keeping to a maximum of two quotes per interpretation to ensure word count is not extended considerably. **Timeline dimension in CSM refers to whether individuals perceive their condition as chronic or acute. Please rephrase your results within this context.**

We have rephrased and re-ordered the timelines dimension section in order to accurately reflect the distinction between chronic or acute as suggested by reviewer 2.  **It seems there are a few interesting results that differentiate between those with and without a illness/condition. This seems rather important; yet, in this version of the manuscript it is not that emphasized.**

The focus of our analysis was to compare the views of young people with and without depression. We have identified all instances where perspectives diverged and identified those perspectives that demonstrated convergence in views. We have edited the results section to ensure this is clear and also provided a summary diagram to further illuminate the key similarities and differences in Figure 1.

**Figure 1:** Overview of the comparative analysis of CYP with and without experience of mental illness.

 **Discussion/Implications

The study has great value, with a very important point, from this reviewer’s point of view, being the high levels of stigma and no sense of recovery in the Indonesian context. What can the authors more specifically suggest for next steps to improve the subjective perspectives of adolescents? Can they develop school-based education/literacy programs? What do these data tell you/inform you about what topics might especially be important to educate individuals on, i.e., cause of mental illness (not a sign of individual weakness).**

We agree with reviewers too and have now made our suggestions for future interventions more explicit in the discussion on page 31:

*These findings also demonstrate a need for and the potential value of implementing mental health literacy interventions in Indonesian community and educational contexts. Such interventions should focus on targeting identified illness beliefs such as individual weakness as a cause of mental illness, the view that mental illnesses are chronic and pervasive the ambivalence towards professional treatment to optimse engagement with and the impact of the intervention.* **Overall, this study has potential. It needs to include more of the published research in the area in more detail. It also needs to add more empirical data elements (actual quotations) to support some of the statements in the results section.**

We have responded to each of the points raised by reviewer 2, adding the suggested additional published research to the introduction and including additional quotations to the results section to support the interpretations presented. We would like to thank this reviewer for their constructive feedback.

**Reviewer #3: In summary, this is an interesting and well-written paper dealing with an important public mental health issue, particularly the focus on young people in LMIC. The rationale for the study is convincing and the methods used are appropriate. However, there are some limitations that should be considered prior to publication:

Major limitations

• There is a methodological inconsistency between the authors’ proposition to focus on narratives and the interviewer’s semi-structured questions. These are different forms of data collection: While narrative approaches refer to biographical experiences and thus, (latent) meanings, semi-structured questions stimulate subjective attitudes or explicit norms. Thus, please check the methodological approach and address the approach and/or the instrument for data collection in more details. Please provide a table including interview prompts and/or questions.**

We are sorry this was not clear in the manuscript. Semi-structured interviews were undertaken and not narrative interviews. It may be that it is the use of the term narrative when participants accounts are described that is introducing confusion. To mitigate against this, we have removed all use of the word narratives from the manuscript. We have also included a list of questions to reassure reviewer 3.

**• Closely related to this, some questions appear as rather closed and/or even suggestive, e.g. “is that a sign that you’re healthy? --- Yes” (line 419-20).**

Semi-structured interviews were undertaken which included a range of open questions with a series of prompts. The use of closed question and prompts within semi-structured interviews are recommended to check understanding on the part of the interviewer and to elucidate in-depth data. We hope this and the response to the point adequately addresses reviewer 3’s points.

**• Some assumptions are presented without sufficient supporting arguments or quotes, e.g. “This was in stark contrast to the time dimensions relating to beliefs about positive mental health and wellbeing” (line 425). Please alaborate on such assumptions and give quotes.**

In line with comments from reviewer 2, we have revisited the manuscript adding additional quotes to support interpretations while trying not to exceed a maximum of two quotes per interpretation so word count did not increase excessively in line with journal requirements.

**• The number of participants is relatively high for an in-depths qualitative study. However, since the findings are presented in the form of examples, it is difficult for readers to assess the dominance (or marginality) of CYPs’ arguments and opinions. Please provide a table including an overview on the themes and the (rough) numbers of relating quotes, e.g. category structure.**

We do not feel our sample size of 43 is particularly large given that we had to purposively sample on a range of different criteria (lived experience, geographical area and gender) and is smaller than other qualitative studies in the global health field. Data saturation was reviewed regularly during the process of data collection and interviews were stopped once there was agreement amongst the team that data saturation had been achieved.

We take reviewer 3’s point about the overview table and acknowledge there is no consensus on the value of such counting in qualitative research. However, we do not feel that a count of quotes relating to themes would be appropriate to our interpretative approach nor would it be consistent with framework analysis (Gale et al., 2013; Suddaby, 2006; Hannah and Lautsch, 2011). Instead, we have instead tried to present the strength of interpreted themes through the use of language. We have ensured that minority views are described as such within the manuscript and that interpretations that were ascribed to by the majority of participants are also identified as such. We have also added additional quotes within the manuscript which we hope reassures reviewer 3.

**• What role does gender play in CYPs’ views on mental health and illness?**

We have now included a sentence in the results section that gender did not appear to have any discernible impact on findings in our study.

**The discussion section appears as relatively unstructured and includes some (new) results.**

- **Please provide a clearer picture of what was found in the study compared to what was found in other studies.**

We have updated the discussion section to ensure each paragraph is comparing study findings to the wider literature. We have also revisited the discussion section and ensured no new results are presented.

- **What kind of differences and similarities were found in comparison with other study settings?**

There has been little research using CSM with young people particularly in low- and middle-income settings. We have now included the two studies that have used this framework in higher income settings and described how our findings support these and also included a discussion on how findings compare with recent evidence from Indonesia which examined perspectives of mental health and illness from the point of view of 16-18 year olds without experience of mental health problems.

**Please provide more insight into the context of the results in terms of cultural aspects. What is rukun?**

We have now defined this within the discussion.

- **I can hardly follow the author’s assumptions about CYP’ coherence (line 544-). Is this a finding? If so, please provide the details in the results section. However, I’m not sure if the method is appropriate to measure coherence.**

We have removed reference to the coherence dimension of the CSM from the discussion.

**• The authors describe that almost 50% of high school student experience depressive symptoms and 10% have a diagnosis. It would be helpful to provide findings from international studies in order to relate these numbers.**

This information is included in the first paragraph of the introduction:

*Globally, between 10-20% of CYP experience mental health problems although the majority go undiagnosed and untreated [2-3].*

**• Different age groups: While in the introductory section, the authors refer to the age group of 15-24, the study group includes CYP between 11 and 15. Please check this and give reasons for the selection of this age group.**

We have now clarified this within the introduction of the manuscript:

*This age range was decided upon following early consultation with Indonesian collaborators because it represents the junior high school population in Indonesia.*

**• Since socio-economic status is known as a significant factor for developing mental health problems, it is surprising that these information is lacking. E.g. parental education or professional status. At least, this limitation should be mentioned and explained in the discussion.**

We have now included an acknowledgement and discussion of this limitation in the discussion.

**• What is the (professional, training) background of the researchers/interviewers?**

We have now included this detail on the methods section:

Interviews ranged between 20 and 40 minutes in length and were undertaken in Bahasa Indonesian by health services researchers at the Ministry of Health in Indonesia or at local sites who received training and ongoing supervision in qualitative research.

**• Please describe the transcription and the translation processes in more detail. What are problems with regard to language translation? How were these problems addressed?**

We have included this additional detail now in the methods section:

*Transcripts were translated into English by a member of the research team. 5% of translated transcripts were checked by a third party not involved in data collection to identify any discrepancies in meaning compared to the original transcript in accordance with the guidelines for undertaking international qualitative research.*

**• What is meant by “marginalised in interviews” (line 303)?**

This has now been defined in the sentence:

*Given the perceived dichotomy between positive mental health and illness, those with mental health problems were often marginalised, treated as insignificant or peripheral, in interviews.*

**• Explanations of what was originally planned (focus groups) is not really important to know for readers (599-602).**

Removed. **Please report your qualitative study in line with standards for reporting qualitative research.**

I hope these revisions meet with your approval and I would like to thank the reviewers for their helpful contributions to the article. Should you have any further queries, please do not hesitate to contact me.

Yours sincerely.

Helen Brooks

Senior Lecturer in Mental Health
